# Incidence, case-fatality rates and risk factors of bloodstream infections caused by *Escherichia coli*, *Klebsiella* species and *Pseudomonas aeruginosa*, England, April 2017 to March 2022

**DOI:** 10.2807/1560-7917.ES.2025.30.17.2400430

**Published:** 2025-05-01

**Authors:** Olisaeloka Nsonwu, Simon Thelwall, Sarah Gerver, Rebecca L Guy, Dimple Chudasama, Russell Hope

**Affiliations:** 1 Antimicrobial Resistance and Healthcare Associated Infections Division, UK Health Security Agency, London, United Kingdom

**Keywords:** Gram-negative, Blood, Antibiotics, Resistance, Case-fatality, Healthcare-associated, Regression, England

## Abstract

**Background:**

*Escherichia coli*, *Klebsiella* spp. and *Pseudomonas aeruginosa* are included in the mandatory surveillance of bloodstream infections (BSI) in England.

**Aim:**

We aimed to investigate the epidemiology of these BSIs in England April 2017–March 2022.

**Methods:**

We extracted data on *E. coli*, *Klebsiella* spp. and *P. aeruginosa* BSI, categorised BSIs as healthcare-associated (HA) or community-associated (CA) and linked to antimicrobial susceptibility and mortality data. We used multivariable logistic regression models to assess predictors of mortality.

**Results:**

The incidence rate of *E. coli* BSI was 71.8 per 100,000 population (95% confidence interval (CI): 71.5–72.1), *Klebsiella* spp. 19.1 (95% CI: 18.9–19.3) and *P. aeruginosa* 7.6 (95% CI: 7.5–7.7). Most (65,467/104,957) BSI episodes were CA. Case-fatality rate was 14.8% (95% CI: 14.6–14.9) in *E. coli*, 20.0% (95% CI: 19.6–20.3) in *Klebsiella* spp. and 25.8% (95% CI: 25.2–26.4) in *P. aeruginosa* BSI. Urinary tract infection (UTI) was the most reported primary infection for *E. coli* (56,961/100,834), *Klebsiella* spp. (9,098/22,827) and *P. aeruginosa* (3,204/8,484) BSI. Insertion or manipulation of urinary catheters was reported for 26.4% (16,136/61,043) of *E. coli*, 41.6% (4,470 /10,734) of *Klebsiella* spp. and 49.0% (2,127/4,341) of *P. aeruginosa* BSI. The adjusted odds ratio (OR) of death among hospital-onset HA-BSI compared to CA-BSI was 2.0 (95% CI: 1.9–2.2) for *E. coli*, 2.1 (95% CI: 2.0–2.3) for *Klebsiella* spp. and 1.7 (95% CI: 1.5–2.0) for *P. aeruginosa*.

**Conclusions:**

Appropriate management of UTIs and urinary catheterisation is essential for reduction of these BSIs.

**Key public health message**

**What did you want to address in this study and why?**

Bloodstream infections (BSI) caused by bacteria of *Escherichia coli*, *Klebsiella* species and *Pseudomonas aeruginosa* can be hard to treat and prolong a patient’s stay in hospital. We describe the spread of these infections in England, the most common factors among cases, and percentage of cases where a patient died between April 2017 and March 2022.

**What have we learnt from this study?**

Bloodstream infections were more common in older patients. A greater percentage of *Klebsiella* species and *P. aeruginosa* BSI were healthcare-associated cases and ended in death compared with *E. coli* BSI. Urinary tract infections were the most common primary focus of infection, and among these, patients from about half of *Klebsiella* species and *P. aeruginosa* BSI cases had a urinary catheter inserted or manipulated.

**What are the implications of your findings for public health?**

Despite some similarities between these infections, different strategies are likely to be required to reduce infection rates. Focusing on older patients with UTI from community settings is likely to yield the greatest reductions in *E. coli* BSI. However, for *Klebsiella* species and *P. aeruginosa* BSI, greater reductions may be achieved by focusing on patients that have had a urinary catheter inserted or manipulated.

## Introduction

Between April 2022 and March 2023, *Escherichia coli*, *Klebsiella* spp. and *Pseudomonas aeruginosa* accounted for 75% of Gram-negative bloodstream infections (GNBSI) and 38% of all bloodstream infections (BSI), excluding those caused by coagulase-negative Staphylococci, in England, Wales and Northern Ireland [1]. Among these, *Klebsiella* spp. and *P. aeruginosa* BSI are more likely to be healthcare-associated (HA), impact patients with weakened immune systems [2-4] and result in high mortality. Consequently, GNBSI lead to lengthy hospital stays and increased costs of care [5-7], thereby constituting a disease burden on patients and health services. Naylor et al. 2019 estimated the annual costs of *E. coli* bacteraemia in England to be Great British Pound (GBP) 14,346,400 (year 2012 GBP value) with an excess cost of GBP 420 (95% confidence interval (CI): GBP 220–630) per patient when associated with third-generation cephalosporin resistance. The burden of these infections on the United Kingdom (UK) population and healthcare services contributed to the ambition of the UK government to halve the number of HA-GNBSI patients identified between April 2016 and March 2017 by 2023–24 [8]. To support this initiative, in April 2017, the mandatory surveillance of *E. coli* BSI in England was expanded to include *Klebsiella* spp. and *P. aeruginosa* BSI.

National Health Service (NHS) acute trusts (secondary care providers) notify all laboratory-confirmed cases of *E. coli*, *Klebsiella* spp. and *P. aeruginosa* BSI to the Healthcare-associated infections (HCAI) Data Capture System (HCAI DCS) of the United Kingdom Health Security Agency (UKHSA). Post-mortem samples are not included. Blood samples are usually collected from patients with symptoms such as persisting fever and abnormal heart rate in line with clinical standards set by NHS England and tested for antimicrobial susceptibility using antimicrobial testing panels based on local recommendations and prescribing policy. In a survey in 2018, 95% of responding laboratories in England (survey response rate 94%) indicated that the European Committee on Antimicrobial Susceptibility Testing (EUCAST; https://www.eucast.org/) or the British Society for Antimicrobial Chemotherapy (BSAC; https://bsac.org.uk/) susceptibility testing breakpoints were used with some variations in testing methods.

In England, between April 2012 and March 2014, genital or urinary tract was the most common primary focus of *E. coli* BSI (41.1%) and 76.1% of the infections were community-onset [9]. In the sentinel study of Abernethy et al. 2017, half of the patients had previous healthcare exposure in the month before the bacteraemia, with antimicrobial therapy and urinary catheterisation reported as common treatments [10]. There are no studies on the mandatory surveillance of *Klebsiella* spp. and *P. aeruginosa* BSI in England.

We aimed to present findings from the first 5 years of the expanded GNBSI national surveillance in England, providing key information on the epidemiology of these infections, and to support the development of infection prevention and control strategies in the UK. In this study, we report the incidence, predisposing risk factors, antimicrobial resistance and 30-day all-cause case-fatality rates and mortality rates associated with these infections.

## Methods

### Data collection

#### Gram-negative bloodstream infections

On 17 October 2022, we extracted data from the UKHSA HCAI DCS [11] on *E. coli*, *Klebsiella* spp. and *P. aeruginosa* BSIs diagnosed by NHS acute trusts in England between 1 April 2017 and 31 March 2022. Data on patient demographics, case and admission details, treatment specialty, predisposing risk factors within 28 days before the specimen date and the suspected primary focus of the infection were extracted. Information on the primary focus of infection is voluntary to include in the report. Multiple risk factors can be reported with each diagnosis. The reported risk factors depend on the primary focus of infection. For example, if the primary focus of infection is a respiratory tract infection, then whether (or not) the patient was intubated or extubated earlier will be reported but not whether the patient had a urinary catheter inserted or manipulated. The opposite is the case when the primary focus of infection is the urinary tract. The applicable risk factors for each primary focus of infection are presented in Supplementary Table 1.

A BSI episode was defined as the first report of a GNBSI from a patient within a fixed 14-day period. Subsequent reports within this fixed period were considered duplicate reports and excluded from all analyses. Episodes of *E. coli*, *Klebsiella* spp. and *P. aeruginosa* BSI were defined separately meaning that a patient could have an episode of any of these pathogens on the same day. Furthermore, the fixed 14-day episode length meant that a patient could have another BSI episode between the 15th and 29th day after the previous BSI episodes. More details can be seen in Supplementary Table 2.

Bloodstream infections were categorised as HA or community-associated (CA). A HA-BSI could be either hospital-onset (HOHA) or community-onset HA (COHA). A HOHA-BSI was one where a blood sample with detection of *E. coli*, *Klebsiella* spp. or *P. aeruginosa* was taken ≥ 3 days after an admission to an NHS acute trust (Box). A COHA-BSI was one where the patient was previously discharged from the same NHS acute trust ≤ 28 days before the specimen date. Any BSI not defined as a HA-BSI was considered a CA-BSI. Information to determine a HA-BSI was only available for BSI episodes after April 2020. Therefore, analyses involving healthcare-association were limited to the period between April 2020 and March 2022.

Box **Definition of healthcare-associated and community-associated bloodstream infections caused by *Escherichia coli*, *Klebsiella* species and *Pseudomonas aeruginosa*, England, April 2020–March 2022**

**Bloodstream infection (BSI):**

• Presence of viable microorganisms in the bloodstream, in this study detection of *Escherichia coli*, *Klebsiella* spp. or *Pseudomonas aeruginosa* from a blood specimen.

**Healthcare-associated BSI (HA-BSI):**

• BSI associated with a healthcare visit.

• *Hospital-onset (HOHA):*

o Blood specimen taken ≥ 3 days after an admission to an NHS acute trust.

• *Community-onset (COHA):*

o BSI is not a HOHA-BSI and patient was previously discharged from the same NHS acute trust ≤ 28 days before the specimen date.

**Community-associated BSI (CA-BSI):**

• Any BSI that was not HA BSI.

#### Mortality

Information on deaths and dates of deaths of the patients with BSIs were derived by deterministic linkage using the patient NHS numbers (unique patient identifier) and dates of birth, to a central repository of patient information (NHS SPINE). We analysed mortality as 30-day all-cause deaths, i.e. deaths from any cause within 30 days of the specimen date. Since a BSI’s episode length was 14 days, a patient could have up to three BSI episodes within this 30-day period. To prevent overestimating the number of deaths, only the most recent BSI episode of each pathogen before the date of death (or the most recent episode of each pathogen for unlinked patients) within the 30-day period was included in all mortality analysis. More details can be seen in Supplementary Table 2.

#### Antimicrobial susceptibility results

Susceptibility results for gentamicin, amikacin, netilmicin, tobramycin, imipenem, cefotaxime ceftazidime, piperacillin-tazobactam, ciprofloxacin and amoxicillin-clavulanic acid were derived by deterministic linkage using the patient’s NHS number and date of birth between the BSI episodes and data from UKHSA’s voluntary laboratory surveillance database Second Generation Surveillance System. If there were different susceptibility results for each antimicrobial within 14 days of the BSI episodes, the most resistant result per antimicrobial was assigned to the episode.

#### Population estimates

England’s mid-year population estimates by age, sex and region for 2017 to 2022 were downloaded from the Office for National Statistics (ONS) website [12], while the quarterly average number of overnight occupied beds and day admissions were sourced from NHS England’s published Bed Availability and Occupancy (KH03) returns [13].

### Data analysis

#### Incidence and mortality rates

Incidence and mortality rates were calculated using the total number of BSI episodes and 30-day all-cause deaths respectively, against England’s mid-year population for all reported episodes or total overnight bed-days and day admissions for HA-BSI. Case-fatality rates (CFR) per pathogen were calculated as the percentage of 30-day all-cause deaths among all BSI episodes included in the mortality analyses. Regional analysis was based on seven NHS administrative regions covering England.

#### Antimicrobial resistance

Antimicrobial resistance was calculated as the percentage of all BSI episodes with susceptibility results that have been assigned resistant (R). This was calculated separately for each antimicrobial or antimicrobial class. In line with EUCAST definitions [14], we considered susceptible (S) and intermediate (I) results (now defined as susceptible with increased exposure) as susceptible. Aminoglycoside resistance was defined as resistance to either gentamicin, amikacin, netilmicin or tobramycin. For *E. coli* and *Klebsiella* spp., carbapenem resistance was defined as resistance to either imipenem or meropenem. For *P. aeruginosa* isolates, carbapenem resistance was defined as resistance to meropenem only. However, we used the result of testing against imipenem as a proxy if there was no susceptibility result for meropenem. Third-generation cephalosporin resistance was defined as resistance to cefotaxime or ceftazidime for *E. coli* and *Klebsiella* spp. isolates but for *P. aeruginosa* isolates, as resistance to ceftazidime only. Multidrug resistance (MDR) for *E. coli* and *Klebsiella* spp. isolates was defined as resistance to any three of the antimicrobial agents or classes: aminoglycosides, piperacillin-tazobactam, third-generation cephalosporin, carbapenems, ciprofloxacin and amoxicillin-clavulanic acid. For isolates of *P. aeruginosa,* susceptibility to amoxicillin-clavulanic acid was not included due to expected intrinsic resistance.

#### Predictors of mortality

Multivariable logistic regression models were used to assess HA-BSI (HOHA and COHA separately) and MDR for increased risk of mortality, expressed as adjusted odds ratios (aOR). Each model was adjusted for patients’ age group, sex, geographical region, year and month of infection as confounding variables. Episodes where data on the outcome and explanatory variables could not be determined were excluded from the regression analysis. We tested for multicollinearity among these variables by calculating scaled generalised variance inflation factors.

Statistical significance was defined as p values ≤ 0.05. Fisher’s exact test p values and Wald 95% confidence intervals (CI) were reported for relative risks (RR). Wald p values and 95% CIs were reported for rate ratios. Wald p values and profile likelihood ratio 95% CIs were reported for the aORs. Scaled GVIF scores of between 1 and 1.5 were interpreted as no considered evidence of multicollinearity. All analyses were done in R version 4.0.2 (https://www.r-project.org/) with the tidyverse, epitools, stats, car and diyar packages [15,16].

## Results

### Gram-negative bloodstream infections

After removing duplicate reports as described in the method section, we identified 277,177 GNBSI episodes among 240,796 patients with BSI between 1 April 2017 and 31 March 2022. There were 202,054 *E. coli*, 53,751 *Klebsiella* spp. and 21,372 *P. aeruginosa* episodes (Table 1). The mean age of the patients was similar across all three pathogens: from 70.4 years (standard deviation (SD): 19.3) in *E. coli* to 67.2 years (SD: 20.8) in *P. aeruginosa*. Male patients had 99,512 (49.3%) episodes of *E. coli,* 33,638 (62.6%) *Klebsiella* spp. and 13,735 (64.3%) *P. aeruginosa*. The overall incidence rates of BSI were 71.8 episodes per 100,000 population (95% CI: 71.5–72.1) for *E. coli*, 19.1 (95% CI: 18.9–19.3) for *Klebsiella* spp. and 7.6 (95% CI: 7.5–7.7) for *P. aeruginosa* (Table 1). The annual incidence rate of *E. coli* BSI declined from 73.6 (95% CI: 72.9–74.4) in 2017–18 to 67.1 (95% CI: 66.4–67.7) in 2021–22. Conversely, over the same period, the incidence rate of *Klebsiella* spp. BSI increased from 17.5 (95% CI: 17.1–17.8) to 20.1 (95% CI: 19.7–20.4). The incidence rate of *P. aeruginosa* BSI was 7.7 (95% CI: 7.5–7.9) in 2017–18 and 7.6 (95% CI: 7.4–7.9) in 2021–22.

**Table 1:** Frequency and incidence rates of *Escherichia coli* (n = 202,054), *Klebsiella* species (n = 53,751) and *Pseudomonas aeruginosa* (n = 21,372) bloodstream infections, England, 1 April 2017–31 March 2022 (n = 240,796 patients)^a^

Characteristics	*Escherichia coli*				*Klebsiella* species				*Pseudomonas aeruginosa*			
	n	%	IR^b^	95% CI	n	%	IR	95% CI	n	%	IR	95% CI
Number of episodes												
April 2017–March 2018	41,020	20.3	73.6	72.9–74.4	9,725	18.1	17.5	17.1–17.8	4,290	20.1	7.7	7.5–7.9
April 2018–March 2019	43,150	21.4	77.0	76.3–77.7	10,632	19.8	19.0	18.6–19.3	4,173	19.5	7.4	7.2–7.7
April 2019–March 2020	43,278	21.4	76.6	75.9–77.4	10,988	20.4	19.5	19.1–19.8	4,314	20.2	7.6	7.4–7.9
April 2020–March 2021	36,683	18.2	64.9	64.2–65.5	11,065	20.6	19.6	19.2–19.9	4,275	20.0	7.6	7.3–7.8
April 2021–March 2022	37,923	18.8	67.1	66.4–67.7	11,341	21.1	20.1	19.7–20.4	4,320	20.2	7.6	7.4–7.9
Total	202,054		71.8	71.5–72.1	53,751		19.1	18.9–19.3	21,372		7.6	7.5–7.7
Age of females (years)												
< 1	1,365	1.3	90.4	85.6–95.3	449	2.2	29.7	27.0–32.6	128	1.7	8.5	7.1–10.1
1–14	578	< 1	2.5	2.3–2.7	300	1.5	1.3	1.1–1.4	258	3.4	1.1	1.0–1.3
15–44	10,997	10.7	20.8	20.4–21.2	2,138	10.6	4.0	3.9–4.2	809	10.6	1.5	1.4–1.6
45–64	18,454	18.0	50.6	49.8–51.3	4,918	24.5	13.5	13.1–13.9	1,806	23.7	4.9	4.7–5.2
65–74	18,957	18.5	131.2	129.3–133.0	4,348	21.6	30.1	29.2–31.0	1,725	22.6	11.9	11.4–12.5
75–84	27,159	26.5	295.6	292.1–299.1	4,624	23.0	50.3	48.9–51.8	1,614	21.2	17.6	16.7–18.4
≥ 85	24,913	24.3	569.1	562.1–576.2	3,310	16.5	75.6	73.1–78.2	1,291	16.9	29.5	27.9–31.1
Total	102,423	50.7	72.0	71.6–72.5	20,087	37.4	14.1	13.9–14.3	7,631	35.7	5.4	5.2–5.5
Age of males (years)												
< 1	2,104	2.1	132.1	126.6–137.9	600	1.8	37.7	34.7–40.8	159	1.2	10.0	8.5–11.7
1–14	478	< 1	2.0	1.8–2.1	401	1.2	1.6	1.5–1.8	313	2.3	1.3	1.1–1.4
15–44	3,857	3.9	7.2	6.9–7.4	1,976	5.9	3.7	3.5–3.8	942	6.9	1.7	1.6–1.9
45–64	18,341	18.4	51.7	50.9–52.4	7,453	22.2	21.0	20.5–21.5	2,834	20.6	8.0	7.7–8.3
65–74	23,038	23.2	171.9	169.7–174.2	7,876	23.4	58.8	57.5–60.1	3,171	23.1	23.7	22.8–24.5
75–84	30,119	30.3	394.4	390.0–398.9	9,309	27.7	121.9	119.4–124.4	3,746	27.3	49.1	47.5–50.7
≥ 85	21,575	21.7	840.9	829.7–852.2	6,023	17.9	234.7	228.8–240.7	2,570	18.7	100.2	96.3–104.1
Total	99,512	49.3	71.5	71.1–72.0	33,638	62.6	24.2	23.9–24.4	13,735	64.3	9.9	9.7–10.0
Healthcare-association^c^	n = 74,094				n = 22,312				n = 8,551			
Healthcare-associated	24,066	32.5	13.2	13.0–13.4	10,568	47.4	5.8	5.7–5.9	4,856	56.8	2.7	2.6–2.7
Community-associated	50,028	67.5	17.8	17.6–17.9	11,744	52.6	4.2	4.1–4.3	3,695	43.2	1.3	1.3–1.4
Primary focus of infection	n = 100,834				n = 22,827				n = 8,484			
Urinary tract	56,961	56.5	NA		9,098	39.9	NA		3,204	37.8	NA	
Hepatobiliary	19,847	19.7			5,244	23.0			533	6.3		
Gastrointestinal (not hepatobiliary)	8,109	8.0			2,117	9.3			687	8.1		
Respiratory tract	7,310	7.2			2,879	12.6			1,523	18.0		
Other	5,699	5.7			2,698	11.8			2,136	25.2		
No underlying focus of infection	2,908	2.9			791	3.5			401	4.7		

BSI: bloodstream infection; CI: confidence interval; IR: incidence rate: NA: not applicable.

**^a^** A patient could have more than one BSI episode of infection.

**^b^** Incidence rate of healthcare-associated BSI is per 100,000 overnight bed-days and day admissions. Incidence rate of hospital-onset BSI is per overnight bed-days. Every other incidence rate is per 100,000 population.

**^c^** Only data on infections between April 2020 and March 2022 were included.

### Demographic distribution

In patients aged ≥ 45 years, incidence rates were higher in males with *Klebsiella* spp. and *P. aeruginosa* BSI (Table 1) than in females. In male patients aged ≥ 85 years, the incidence rate ratio (IRR) compared to females with *E. coli* was 1.48 (95% CI: 1.45–1.50, p < 0.01), while IRR for *Klebsiella* spp. was 3.1 (95% CI: 3.0–3.2; p < 0.01) and 3.4 for *P. aeruginosa* (95% CI: 3.2–3.6; p < 0.01). The incidence rate of *E. coli* BSI in females aged 15–44 years was higher than in males (IRR = 2.9; 95% CI: 2.8–3.0; p < 0.01). The geographical distribution of incidence rates of HA-BSIs and CA-BSIs between 2020–21 and 2021–22 is presented in Supplementary Table 5.

### Healthcare-association

Between 1 April 2020 and 31 March 2022, most (62.4%; 65,467/104,957) *E. coli*, *Klebsiella* spp. and *P. aeruginosa* BSI episodes were CA. However, 32.5% (24,066/74,094) of *E. coli*, 47.4% (10,568/22,312) of *Klebsiella* spp. and 56.8% (4,856/8,551) of *P. aeruginosa* BSI episodes were HA (Table 1). The incidence rate for HA-BSI of *E. coli*, *Klebsiella* spp. and *P. aeruginosa* was 13.2 (95% CI: 13.0–13.4), 5.8 (95% CI: 5.7–5.9) and 2.7 (95% CI: 2.6–2.7) episodes per 100,000 overnight bed-days and day admissions, respectively. Of the CA-BSIs, 6.8% (n = 7,165) had a predisposing risk factor based on the reported primary focus of infection: 6.8% of *E. coli* (n = 4,970), 6.8% of *Klebsiella* spp. (n = 1,512) and 8% of *P. aeruginosa* (n = 683). The predisposing risk factors are detailed in Supplementary Table 1.

### Primary focus of infection and predisposing risk factors

When information was available among GNBSI episodes between 1 April 2017 and 31 March 2022, urinary tract infections were the most reported primary infections: 56.5% (56,961/100,834) of *E. coli*, 39.9% (9,098/22,827) of *Klebsiella* spp. and 37.8% (3,204/8,484) of *P. aeruginosa* BSI episodes. Respiratory tract infection (RTI) was more common in *P. aeruginosa* (18.0%; 1,523/8,484) than in *E. coli* (7.2%; 7,310/100,834) or *Klebsiella* spp. (12.6%; 2,879/22,827) BSI. More information can be seen in Supplementary Figures 1-2 and Supplementary Table 4.

Among all 277,177 GNBSI episodes, 141,131 (50.9%) were reported with information on predisposing risk factors within 28 days prior to the episode. The most reported risk factors based on the primary focus of infection were the insertion or manipulation of urinary catheters or vascular devices. Information on insertion or manipulation of urinary catheters was reported for 26.4% (16,136/61,043) of *E. coli,* 41.6% (4,470/10,734) of *Klebsiella* spp. and 49.0% (2,127/4,341) of *P. aeruginosa* episodes. Vascular devices were inserted in 27.4% (3,926/14,324) *E. coli*, 49.1% (2,224/4,526) *Klebsiella* spp. and 52.8% (1,266/2,396) *P. aeruginosa* episodes. During the study period, information on urinary catheterisation was known for 72% of *E. coli*, 62% of *Klebsiella* spp. and 55% of *P. aeruginosa* BSI with a UTI primary focus of infection (or none at all).

### Antimicrobial resistance

Between 1 April 2020 and 31 March 2022, we received AMR data for 90.4% (67,465/74,606) of *E. coli* isolates, 89.3% (20,008/22,406) of *Klebsiella* spp. isolates and 89.7% (7,711/8,595) of *P. aeruginosa* isolates. Of the *P. aeruginosa* isolates from patients*,* 6.1% (461/7,558; 95% CI: 5.6–6.7%) were carbapenem resistant compared to < 1% of *Klebsiella* spp. and *E. coli* isolates (Table 2). Resistance to amoxicillin-clavulanic acid was 41.9% (27,135/64,693; 95% CI: 41.6–42.3%) in *E. coli* isolates and 29.9% (5,754/19,235; 95% CI: 29.3–30.6%) in *Klebsiella* spp. isolates. Resistance to third-generation cephalosporins was 11.6% (6,546/56,657; 95% CI: 11.3–11.8%) in *E. coli*, 13.4% (2,268/16,897; 95% CI: 12.9–13.9%) in *Klebsiella* spp. and 6.6% (497/7,494; 95% CI: 6.1–7.2%) in *P. aeruginosa* isolates. Resistance to piperacillin-tazobactam was 9.6% (6,007/62,749; 95% CI: 9.3–9.8%) in *E. coli*, 16.3% (3,063/18,779; 95% CI: 15.8–16.8%) in *Klebsiella* spp. and 8.0% (580/7,287; 95% CI: 7.3–8.6%) in *P. aeruginosa* isolates. Antimicrobial resistance was generally higher in isolates from HA-BSI cases compared with those from CA-BSI cases (Table 2). Of the 66,617 tested isolates of *E. coli,* 10.5% (n = 7,003; 95% CI: 10.3–10.7) were MDR, versus 11.6% (2,294/19,780; 95% CI: 11.2–12.1) of *Klebsiella* spp. isolates and 3.6% (276/7,608; 95% CI: 3.2–4.1) of *P. aeruginosa* isolates. Isolates from HA-BSI were more resistant. For example, 14.9% (3,215/21,582; 95% CI: 14.4–15.4) of *E. coli*, 16.4% (1,528/9,338; 15.6–17.1) of *Klebsiella* spp. and 5.2% (225/4,296; 95% CI: 4.6–5.9) of *P. aeruginosa* isolates were MDR.

**Table 2:** Antimicrobial resistance in *Escherichia coli* (n = 67,465), *Klebsiella* species (n = 20,008) and *Pseudomonas aeruginosa* (n = 7,711) bloodstream infections in England, by healthcare or community association, England, 1 April 2020–31 March 2022^a^

Healthcare-association	*Escherichia coli*				*Klebsiella* species					*Pseudomonas aeruginosa*				
	Tested		Resistant		Tested		Resistant			Tested		Resistant		
	n	%	%	95% CI	n	%	%	95% CI		n	%	%		95% CI
Amoxicillin-clavulanic acid														
Total	64,693	86.7	41.9	41.6–42.3	19,235	85.8	29.9	29.3–30.6		NA		NA		
HA	20,904	86.9	49.8	49.1–50.5	9,059	85.7	37.5	36.5–38.5						
CA	43,333	86.6	38.2	37.7–38.6	10,093	85.9	23.1	22.3–24.0						
Aminoglycosides														
Total	67,113	90.0	11.2	11.0–11.5	19,895	88.8	7.9	7.5–8.3		7,291	84.8	4.2	3.7–4.7	
HA	21,701	90.2	15.1	14.7–15.6	9,362	88.6	10.3	9.7–10.9		4,124	84.9	4.7	4.0–5.3	
CA	44,948	89.8	9.4	9.1–9.6	10,449	89.0	5.7	5.3–6.2		3,130	84.7	3.6	3.0–4.3	
Ciprofloxacin														
Total	65,552	87.9	17.3	17.0–17.6	19,442	86.8	11.3	10.9–11.8		7,593	88.3	7.4	6.8–8.0	
HA	21,207	88.1	22.3	21.7–22.8	9,169	86.8	14.7	14.0–15.4		4,293	88.4	8.9	8.1–9.8	
CA	43,886	87.7	14.8	14.5–15.2	10,191	86.8	8.3	7.8–8.9		3,263	88.3	5.5	4.7–6.3	
Carbapenems^b^														
Total	65,997	88.5	0.2	0.1–0.2	19,413	86.6	0.6	0.5–0.7		7,558	87.9	6.1	5.6–6.7	
HA	21,401	88.9	0.2	0.2–0.3	9,145	86.5	1.0	0.8–1.3		4,271	88.0	8.8	8.0–9.7	
CA	44,141	88.2	0.2	0.1–0.2	10,185	86.7	0.3	0.2–0.4		3,249	87.9	2.5	2.0–3.1	
Third-generation cephalosporins^c^														
Total	56,657	75.9	11.6	11.3–11.8	16,897	75.4	13.4	12.9–13.9		7,494	87.2	6.6	6.1–7.2	
HA	18,187	75.6	15.4	14.9–16.0	7,952	75.2	18.6	17.8–19.5		4,245	87.4	9.3	8.4–10.2	
CA	38,042	76.0	9.7	9.4–10.0	8,868	75.5	8.8	8.2–9.4		3,212	86.9	3.1	2.6–3.8	
Piperacillin–tazobactam														
Total	62,749	84.1	9.6	9.3–9.8	18,779	83.8	16.3	15.8–16.8		7,287	84.8	8.0	7.3–8.6	
HA	20,434	84.9	13.3	12.9–13.8	8,893	84.2	22.2	21.3–23.1		4,138	85.2	10.9	10.0–11.9	
CA	41,860	83.7	7.7	7.5–8.0	9,806	83.5	11.0	10.4–11.7		3,113	84.2	4.0	3.4–4.8	
Multidrug resistance^d^														
Total	66,617	89.3	10.5	10.3–10.7	19,780	88.3	11.6		11.2–12.1	7,608	88.5	3.6	3.2–4.1	
HA	21,582	89.7	14.9	14.4–15.4	9,338	88.4	16.4		15.6–17.1	4,296	88.5	5.2	4.6–5.9	
CA	44,575	89.1	8.4	8.1–8.6	10,359	88.2	7.3		6.8–7.9	3,275	88.6	1.5	1.1–2.0	

BSI: bloodstream infection; CA: community-associated bloodstream infection; CI: confidence interval; *E.*: *Escherichia*; HA: healthcare-associated bloodstream infection; NA: not applicable; *P.*: *Pseudomonas*.

^a^ Includes 466 *E. coli*, 84 *Klebsiella* spp. and 38 *P. aeruginosa* BSI cases that could not be classified as CA or HA due to missing data on discharge prior to the case.

^b^ For *P. aeruginosa* isolates, carbapenem resistance was defined as resistance to meropenem only however, an imipenem result was used as a proxy if there was no susceptibility result for meropenem.

^c^ Third-generation cephalosporin resistance was defined as resistance to cefotaxime or ceftazidime for *E. coli* and *Klebsiella* spp. isolates but was defined as resistance to ceftazidime only for *P. aeruginosa* isolates.

^d^ Multidrug resistance (MDR) for *E. coli* and *Klebsiella* spp. isolates was defined as resistance to any three of the following antimicrobial agents or classes: aminoglycosides, piperacillin-tazobactam, third-generation cephalosporin, carbapenems, ciprofloxacin and amoxicillin-clavulanic acid. For *P. aeruginosa,* the same definition was applied, except for susceptibility to amoxicillin-clavulanic acid which was not considered.

### Mortality and case-fatality

Information on patient death was not known for 7,561 (2.7%) of 277,177 episodes. Additionally, 1,450 episodes within the 30-day period for all-cause deaths were excluded from the analyses to avoid over reporting the number of deaths. Case-fatality rate of *E. coli* BSI was 14.8% (29,036/196,384; 95% CI: 14.6–14.9%), lower than *Klebsiella* spp. or *P.* *aeruginosa* BSI (Table 3). However, the highest mortality rate (10.3 deaths per 100,000 population-years; 95% CI: 10.2–10.4) was in *E. coli* BSI (Table 3). Conversely, CFR for *P. aeruginosa* BSI was higher (5,347/20,755; 25.8%; 95% CI: 25.2–26.4%) but the mortality rate was lower (1.9 deaths per 100,000 population-years; 95% CI: 1.9–2.0%). Among patients aged ≥ 15 years with *E. coli* and *Klebsiella* spp. BSI, CFR increased with age (Table 3). The largest differences in CFRs between sexes were in patients with *E. coli* BSI who were aged 15–44 years (RR = 3.5; 95% CI: 3.0–4.1; p < 0.01). The RRs of CFR in HA-BSI compared with CA-BSI were 1.6 (95% CI: 1.5–1.6; p < 0.01) for *E. coli*, 1.4 (95% CI: 1.4–1.5; p < 0.01) for *Klebsiella* spp. and 1.2 (95% CI: 1.1–1.3; p < 0.01) for *P. aeruginosa*. The RR of CFR in MDR-BSI compared with non-MDR-BSI was 1.2 (95% CI: 1.1–1.2; p < 0.01) for *E. coli*, 1.1 (95% CI: 1.0–1.2; p < 0.01) for *Klebsiella* spp. and 1.0 (RR: 1.0; 95% CI; 0.9–1.3: p = 0.7) for *P. aeruginosa* BSI cases.

**Table 3:** Case-fatality rate and mortality rates of *Escherichia coli* (n = 29,036), *Klebsiella* spp. (n = 10,396). and *Pseudomonas aeruginosa* (n = 5,347) bloodstream infections within 30 days of sampling, England, 1 April 2017–31 March 2022

Characteristics	*Escherichia coli*					*Klebsiella* species					*Pseudomonas aeruginosa*				
	n	MR^a^	95% CI	CFR%	95% CI	n	MR	95% CI	CFR%	95% CI	n	MR	95% CI	CFR%	95% CI
Total deaths	29,036	10.3	10.2–10.4	14.8	14.6–14.9	10,396	3.7	3.6–3.8	20.0	19.6–20.3	5,347	1.9	1.9–2.0	25.8	25.2–26.4
Number of deaths															
April 2017–March 2018	5,887	10.6	10.3–10.8	14.8	14.5–15.2	1,896	3.4	3.3–3.6	20.3	19.5–21.1	1,121	2.0	1.9–2.1	27.0	25.7–28.4
April 2018–March 2019	5,825	10.4	10.1–10.7	13.9	13.6–14.2	1,934	3.5	3.3–3.6	18.8	18.0–19.5	977	1.7	1.6–1.9	24.2	22.9–25.5
April 2019–March 2020	6,017	10.7	10.4–10.9	14.3	14.0–14.7	2,048	3.6	3.5–3.8	19.2	18.5–20.0	1,034	1.8	1.7–1.9	24.7	23.4–26.0
April 2020–March 2021	5,758	10.2	9.9–10.4	16.1	15.7–16.5	2,357	4.2	4.0–4.3	22.0	21.2–22.7	1,159	2.0	1.9–2.2	27.8	26.4–29.2
April 2021–March 2022	5,549	9.8	9.6–10.1	15.0	14.7–15.4	2,161	3.8	3.7–4.0	19.6	18.9–20.4	1,056	1.9	1.8–2.0	25.1	23.8–26.4
Total	29,036	10.3	10.2–10.4	14.8	14.6–14.9	10,396	3.7	3.6–3.8	20.0	19.6–20.3	5,347	1.9	1.9–2.0	25.8	25.2–26.4
Age of females (years)															
< 1	87	5.8	4.6–7.1	8.4	6.8–10.3	39	2.6	1.8–3.5	10.2	7.3–13.6	25	1.7	1.1–2.4	25.0	16.9–34.7
1–14	15	0.1	< 0.1–0.1	2.8	1.6–4.6	4	< 0.1	< 0.1– < 0.1	1.4	< 1–3.7	15	0.1	< 0.1–0.1	6.1	3.5–9.9
15–44	280	0.5	0.5–0.6	2.7	2.4–3.0	167	0.3	0.3–0.4	8.1	7.0–9.4	95	0.2	0.1–0.2	12.2	10.0–14.7
45–64	1,842	5.0	4.8–5.3	10.3	9.9–10.7	794	2.2	2.0–2.3	16.6	15.6–17.7	430	1.2	1.1–1.3	24.7	22.6–26.7
65–74	2,268	15.7	15.1–16.4	12.3	11.8–12.8	889	6.2	5.8–6.6	20.9	19.7–22.2	532	3.7	3.4–4.0	31.5	29.3–33.8
75–84	3,774	41.1	39.8–42.4	14.2	13.8–14.6	1,058	11.5	10.8–12.2	23.4	22.2–24.7	558	6.1	5.6–6.6	35.4	33.0–37.8
≥ 85	4,811	109.9	106.8–113.1	19.7	19.2–20.2	966	22.1	20.7–23.5	29.8	28.3–31.5	521	11.9	10.9–13.0	41.3	38.5–44.0
Total	13,077	9.2	9.0–9.4	13.1	12.9–13.4	3.917	2.8	2.7–2.8	20.1	19.5–20.7	2,176	1.5	1.5–1.6	29.4	28.4–30.5
Age of males (years)															
< 1	147	9.2	7.8–10.9	8.4	7.2–9.8	46	2.9	2.1–3.9	9.6	7.1–12.7	49	3.1	2.3–4.1	38.0	29.6–46.9
1–14	16	0.1	< 0.1–0.1	3.7	2.1–5.9	17	0.1	< 0.1–0.1	4.5	2.7–7.2	14	0.1	< 0.1–0.1	4.9	2.7–8.1
15–44	343	0.6	0.6–0.7	9.3	8.4–10.3	216	0.4	0.3–0.5	11.7	10.3–13.3	120	0.2	0.2–0.3	13.5	11.3–15.9
45–64	2,301	6.5	6.2–6.8	12.9	12.4–13.4	1,296	3.7	3.5–3.9	18.0	17.1–18.9	605	1.7	1.6–1.8	21.9	20.4–23.5
65–74	3,171	23.7	22.8–24.5	14.1	13.7–14.6	1,441	10.8	10.2–11.3	18.8	17.9–19.7	792	5.9	5.5–6.3	25.6	24.0–27.1
75–84	5,123	67.1	65.3–69.0	17.4	16.9–17.8	1,885	24.7	23.6–25.8	20.7	19.9–21.5	898	11.8	11.0–12.6	24.5	23.1–25.9
≥ 85	4,845	188.8	183.5–194.2	22.9	22.4–23.5	1,575	61.4	58.4–64.5	26.8	25.6–27.9	691	26.9	25.0–29.0	27.5	25.7–29.2
Total	15,946	11.5	11.3–11.6	16.5	16.2–16.7	6,476	4.7	4.5–4.8	19.9	19.5–20.3	3,169	2.3	2.2–2.4	23.7	23.0–24.5
Healthcare-association^b^															
Healthcare-associated	4,836	2.7	2.6–2.7	20.6	20.1–21.1	2,540	1.4	1.3–1.4	24.8	24.0–25.7	1,360	0.7	0.7–0.8	28.8	27.5–30.1
Community-associated	6,391	2.3	2.2–2.3	13.1	12.8–13.4	1,958	0.7	0.7–0.7	17.1	16.5–17.8	848	0.3	0.3–0.3	23.4	22.1–24.8
Total	11,227					4,498					2,208				
Primary focus of infection															
Urinary tract	5,421			9.8	9.6–10.0	1,193			13.5	12.8–14.3	465			14.9	13.6–16.2
Hepatobiliary	2,739			14.2	13.7–14.7	916			18.0	17.0–19.1	159			30.5	26.5–34.6
Gastrointestinal (not hepatobiliary)	1,750			22.3	21.4–23.3	526			25.7	23.8–27.6	184			27.6	24.2–31.1
Respiratory tract	1,946			27.4	26.4–28.5	958			34.4	32.7–36.2	675			45.4	42.8–47.9
Other	789			14.3	13.4–15.2	362			13.9	12.6–15.3	397			19.3	17.6–21.1
No underlying focus of infection	536			19.3	17.8–20.8	184			24.4	21.4–27.7	130			34.1	29.4–39.1
Total	13,181					4,139					2,010				

BSI: bloodstream infection; CFR: case fatality rate; CI: confidence interval; MR: mortality rate.

**^a^** Mortality rate of healthcare-associated BSI is per 100,000 overnight bed days and day-admissions. Incidence rate of hospital-onset BSI is per overnight bed days. Every other mortality rate is per 100,000 population.

**^b^** Only data on infections between April 2020 and March 2022 were included.

### Predictors of mortality

We included 65,915 (88.5%) *E. coli*, 19,617 (87.7%) *Klebsiella* spp. and 7,551 (88.0%) *P. aeruginosa* episodes of BSI between 1 April 2020 and 31 March 2022 in the logistic regression analysis. We found no evidence of considerable multicollinearity from the scaled generalised inflation factors for the variables included in the regression model, more details can be seen in Supplementary Table 3. After adjusting for confounders, the aOR of death in HOHA-BSI and COHA-BSI compared with CA-BSI was 2.0 (95% CI: 1.9–2.2; p < 0.01) and 1.4 (95% CI 1.4–1.5; p < 0.01) for *E. coli*, 2.1 (95% CI: 2.0–2.3, p < 0.01) and 1.4 (95% CI: 1.2–1.5, p < 0.01) for *Klebsiella* spp., and 1.7 (95% CI: 1.5–2.0, p < 0.01) and 1.3 (95% CI: 1.1–1.6, p < 0.01) for *P. aeruginosa* (Table 4). The odds of death with MDR-BSI compared with non-MDR-BSI were not statistically significant (*E. coli*: aOR = 1.1; 95% CI: 1.0–1.1; p = 0.1; *Klebsiella* spp.: aOR = 1.0; 95% CI: 0.9–1.1; p = 0.7 and *P. aeruginosa:* aOR = 1.2; 95% CI: 0.9–1.6; p = 0.2).

**Table 4:** Logistic regression analysis for predictors of mortality of *Escherichia coli* (n = 65,915), *Klebsiella* spp. (n = 19,617) and *Pseudomonas aeruginosa* (n = 7,551) bloodstream infections, England, 1 April 2020–31 March 2022

Characteristics	*Escherichia coli*			*Klebsiella* species			*Pseudomonas aeruginosa*		
	aOR	95% CI	p value	aOR	95% CI	p value	aOR	95% CI	p value
Age (years)									
1–14	1.0	Reference							
< 1	2.8	1.4–6.4	< 0.01	3.6	1.6–9.6	< 0.01	9.6	3.9–27.4	< 0.01
15–44	2.7	1.4–6.0	< 0.01	4.5	2.1–11.5	< 0.01	4.5	2.1–11.8	< 0.01
45–64	6.3	3.3–13.9	< 0.01	9.3	4.5–23.6	< 0.01	9.4	4.5–24.1	< 0.01
65–74	7.4	3.9–16.2	< 0.01	11.3	5.5–28.8	< 0.01	13.9	6.7–35.5	< 0.01
75–84	9.1	4.8–20.1	< 0.01	12.2	5.9–31.1	< 0.01	15.1	7.3–38.8	< 0.01
≥ 85	12.8	6.8–28.2	< 0.01	19.5	9.4–49.8	< 0.01	18.7	8.9–47.9	< 0.01
Sex									
Female	1.0	Reference							
Male	1.2	1.1–1.3	< 0.01	1.0	0.9–1.1	1.0	0.8	0.7–0.8	< 0.01
Multidrug resistance									
MDR	1.1	1.0–1.1	0.1	1.0	0.9–1.1	0.7	1.2	0.9–1.6	0.2
Healthcare-association									
CA	1.0	Reference							
COHA	1.4	1.4–1.5	< 0.01	1.4	1.2–1.5	< 0.01	1.2	1.1–1.4	< 0.01
HOHA	2.0	1.9–2.2	< 0.01	2.1	2.0–2.3	< 0.01	1.7	1.5–2.0	< 0.01

aOR: adjusted odds ratio; CA: community-associated; CI: confidence interval; COHA: community-onset healthcare-associated; HOHA: hospital-onset healthcare-associated; MDR: multidrug resistance.

## Discussion

We described the epidemiology of the three most common GNBSI in England: UKHSA routinely compares GNBSI reports with data on isolates from UKHSA’s Second Generation Surveillance System – a voluntary laboratory-based surveillance scheme, to monitor and follow up on missed cases. Between April 2019 and March 2020, it was estimated that 89%, 97%, 94% of all *E. coli*, *Klebsiella* spp. and *P. aeruginosa* BSI cases in England were notified to the HCAI DCS [17]. Most cases were captured by the surveillance scheme and therefore included in our study. We considered the likelihood of sampling bias as low because of the mandatory requirement to notify these GNBSI episodes.

*Escherichia coli* was the most common cause of BSI. The annual incidence of *E. coli* BSI declined during the study period, mostly between April 2019 and March 2021 during the COVID-19 pandemic. Cancelled or delayed elective procedures during the pandemic [18] likely contributed to this reduction but investigations to better understand this reduction are ongoing. Between 2016 and 2018, fewer *E. coli* BSI cases were notified [17]. The exact cause of this is not yet known but increased awareness of GNBSIs following the introduction of the UK’s 2016 ambition to half the number of HA-GNBSI may have improved infection control practices. Conversely, incidence of all *Klebsiella* spp. increased in the period 2017–22. Sloot et al. (2022) showed that during the pandemic, HOHA-BSI cases of *Klebsiella* spp. and *P. aeruginosa* were also positive for severe acute respiratory syndrome coronavirus-2 (SARS-CoV-2) [19].

Like in other studies, most of the infections in our study were CA [9,20], most older patients were associated with UTI [20,21], and HA-BSI cases had higher CFR [21].

The epidemiology of the three infections differs, pointing towards the need for separate approaches and focus areas to decrease the infections. A greater proportion of *E. coli* BSI episodes were CA compared with *Klebsiella* spp. and *Pseudomonas aeruginosa*. The proportion of CA *Klebsiella* spp. and *P. aeruginosa* BSI has increased over time in the UK [21,22]. In the studies of Livermore et al. 2008a and Livermore et al. 2008b, 39.6% *Klebsiella* spp. and 33% of *P. aeruginosa* BSI were community-onset BSI (combination of CA-BSI and COHA-BSI) compared with 52.6% and 43.2% being CA-BSI in our study. Although there were differences between these studies, this change could demonstrate the importance of non-hospital settings, such as care homes or homes for these infections, as some invasive procedures are increasingly managed in these settings [23]. In a study on the use of catheters in patients receiving NHS-funded care, about a quarter of all patients with urinary catheters were from non-hospital settings [24]. In our study, about half of all *Klebsiella* spp. and *P. aeruginosa* BSI with a UTI as a primary focus of infection were in patients with a urinary catheter inserted or manipulated within a month before. Reporting information on the insertion and manipulation of a urinary catheter (as well as other predisposing risk factors) to the HCAI DCS is voluntary [11] and therefore limits the generalisability of our finding.

The CFR in *P. aeruginosa* BSI (25.8%; 95% CI: 25.2–26.4%) was higher than in *E. coli* (14.8%; 95% CI: 14.6–14.9) and *Klebsiella* spp. (20.0%; 95% CI: 19.6–20.3). However, the aOR in HA-BSI relative to CA-BSI was lowest in *P. aeruginosa* BSI cases.

Except for carbapenem resistance, resistance to the tested antimicrobial agents was greater in *E. coli* and *Klebsiella* spp. isolates compared with *P. aeruginosa*. Overall, resistance was higher in HA-BSI cases. This is not surprising given the higher selection pressure for resistant strains in healthcare settings due to greater antimicrobial use [25]. Interpreting the burden of AMR infections can be challenging due to factors such as local antimicrobial resistance and prescription rates, the burden of resistant strains, availability of alternative treatment options and MDR [26]. In our study, to mitigate this, MDR was used to assess burden, where it was found not to be a predictor of mortality. However, caution should be taken in interpreting this as evidence of no impact of AMR on mortality as we did not have information on appropriate antimicrobial therapy. In other studies, inappropriate antimicrobial therapy has had varying impact on the risk of mortality [27,28]. Future work will investigate the impact of AMR and appropriate prescribing on mortality and other patient outcomes. Exclusion of cases with unknown MDR result might have affected the results but we found no statistically significant difference in odds of death between MDR and non-MDR-GNBSI compared with those with unknown MDR data.

Surveillance of GNBSI is essential to developing and implementing prevention and control strategies in the UK and supporting the UK’s national action plan (NAP) for antimicrobial resistance [29]. Reducing AMR and incidence of GNBSI is one of the outcomes of the UK NAP. It is important that continued GNBSI surveillance is robust enough to provide insights on groups with the greatest burden of infection and help target interventions.

## Conclusion

Bloodstream infections caused by Gram-negative bacteria are severe infections which can be fatal. Most BSIs in our study were community-associated and urinary tract infection was the most common primary focus of infection. To reduce GNBSI, appropriate management of UTIs and stringent hygienic routines in catheterisation are necessary.

## Supplementary Data

474000 Supplementary Material
