# Supplementary material for: Incidence, case-fatality rates and risk factors of bloodstream infections caused by Escherichia coli, Klebsiella species and Pseudomonas aeruginosa, England, April 2017 to March 2022
Source: Euro Surveill. 2025 May 1;30(17):2400430. doi: 10.2807/1560-7917.ES.2025.30.17.2400430 (PMC12046971; doi:10.2807/1560-7917.ES.2025.30.17.2400430)

Supplementary materials

This supplementary material is hosted by Eurosurveillance as supporting information alongside the article "Incidence, case-fatality rates and risk factors of bloodstream infections caused by *Escherichia coli*, *Klebsiella* species and *Pseudomonas aeruginosa*, England, April 2017 to March 2022", on behalf of the authors, who remain responsible for the accuracy and appropriateness of the content. The same standards for ethics, copyright, attributions and permissions as for the article apply. Supplements are not edited by Eurosurveillance and the journal is not responsible for the maintenance of any links or email addresses provided therein

Table 1: Relevant predisposing risk factors for each reported source of infection

Primary focus of infection	Predisposing risk factors
Cardiovascular or Vascular (without prosthetic material, including fistula infection), Cardiovascular or Vascular (with prosthetic material e.g. EVAR, stent, valve, prosthetic fistula), Central Nervous System and Gastrointestinal or Intra-abdominal collection (excluding hepatobiliary)	Absolute Neutrophil count less than 500 (0.5) at time and Surgery (30 days or 12 months prosthetic material) prior
No underlying focus of infection	Absolute Neutrophil count less than 500 (0.5) at time, Diabetic foot ulcer or infection 28 days prior, Hepatobiliary procedure (ERCP, MRCP or similar) 28 days prior, Intubated (ET or PT) or extubated 28 days prior, Open wounds or ulcer (excluding diabetic foot infection) 28 days prior, Surgery (30 days or 12 months prosthetic material) prior, Urinary catheter (including intermittent or temporary) in last 28 days prior, Urinary catheter (including intermittent or temporary) manipulated 28 days prior, UTI treatment 28 days prior to onset, Vascular device (PPM or ICD) or CVC inserted, removed, manipulated 28 days prior and Prostate biopsy in 28 days prior
Bone and Joint (no prosthetic material), Bone and Joint (with prosthetic material) and Skin or Soft Tissue (including ulcers, cellulitis, diabetic foot infections without OM)	Absolute Neutrophil count less than 500 (0.5) at time, Diabetic foot ulcer or infection 28 days prior, Open wounds or ulcer (excluding diabetic foot infection) 28 days prior and Surgery (30 days or 12 months prosthetic material) prior
Upper Respiratory Tract and ENT	Absolute Neutrophil count less than 500 (0.5) at time, Intubated (ET or PT) or extubated 28 days prior and Surgery (30 days or 12 months prosthetic material) prior
Lower Urinary Tract and Upper Urinary Tract (pyelonephritis/abscess)	Absolute Neutrophil count less than 500 (0.5) at time, Surgery (30 days or 12 months prosthetic material) prior, Urinary catheter (including intermittent or temporary) in last 28 days prior, Urinary catheter (including intermittent or temporary) manipulated 28 days prior, UTI treatment 28 days prior to onset and Prostate biopsy in 28 days prior
Hepatobiliary	Hepatobiliary procedure (ERCP, MRCP or similar) 28 days prior, Surgery (30 days or 12 months prosthetic material) prior and Absolute Neutrophil count less than 500 (0.5) at time
Lower Respiratory Tract (pneumonia, VAP, bronchiectasis, exacerbation of COPD etc)	Intubated (ET or PT) or extubated 28 days prior, Absolute Neutrophil count less than 500 (0.5) at time and Surgery (30 days or 12 months prosthetic material) prior
Genital system (including prostate if male)	Urinary catheter (including intermittent or temporary) in last 28 days prior, Urinary catheter (including intermittent or

	temporary) manipulated 28 days prior, UTI treatment 28 days prior to onset, Prostate biopsy in 28 days prior, Surgery (30 days or 12 months prosthetic material) prior and Absolute Neutrophil count less than 500 (0.5) at time
No clinical signs of infection and Unknown	Urinary catheter (including intermittent or temporary) in last 28 days prior, Urinary catheter (including intermittent or temporary) manipulated 28 days prior, UTI treatment 28 days prior to onset, Prostate biopsy in 28 days prior, Vascular device (PPM or ICD) or CVC inserted, removed, manipulated 28 days prior, Intubated (ET or PT) or extubated 28 days prior, Surgery (30 days or 12 months prosthetic material) prior, Hepatobiliary procedure (ERCP, MRCP or similar) 28 days prior, Open wounds or ulcer (excluding diabetic foot infection) 28 days prior, Diabetic foot ulcer or infection 28 days prior and Absolute Neutrophil count less than 500 (0.5) at time
Intravascular device (including Pacemaker/ICD or CVC)	Vascular device (PPM or ICD) or CVC inserted, removed, manipulated 28 days prior, Absolute Neutrophil count less than 500 (0.5) at time and Surgery (30 days or 12 months prosthetic material) prior

Table 2: An illustration of the 14-day fixed episode-window and 30-day death window deduplication methods.

Case deduplication

Number of days from the earliest BSI's specimen date	1	5	10	14	15	20	25	29	30	35	40	43 (Death)
	(grey)	(light green)	(light green)	(light green)	(grey)	(light green)	(light green)	(grey)	(light green)	(light green)	(light green)	(grey)

Thirty-day death window deduplication

Number of days from the earliest BSI's specimen date	1	5	10	14	15	20	25	29	30	35	40	43 (Death)
	(grey)	(light green)	(light green)	(orange)	(orange)	(orange)	(orange)	(orange)	(orange)	(orange)	(orange)	(black)

(grey)	Index BSI included in this study.
(light green)	Duplicate BSI excluded from this study.
(black)	Index BSI within 30 days of death included in case fatality rate, mortality rate and predictors of mortality analyses
(orange)	Duplicate BSI within 30 days of death excluded from case fatality rate, mortality rate and predictors of mortality analyses.

Table 3: Generalised Variance Inflation Factors for all variables in Logistic Regression.

**Organism**	**Variable**	**Generalised Variance Inflation Factor (GVIF)**	**Degree of Freedom (df)**	**Scaled Generalised Variance Inflation Factor GVIF^(1/(2*Df))**
E. coli	Multi-drug resistance	1.03	1	1.02
E. coli	Healthcare-association	1.04	2	1.01
E. coli	Sex	1.02	1	1.01
E. coli	Age group (years)	1.04	6	1.00
E. coli	Region	1.06	41	1.00
E. coli	Financial year	1.01	1	1.00
E. coli	Month	1.02	11	1.00
Klebsiella spp.	Multi-drug resistance	1.04	1	1.02
Klebsiella spp.	Healthcare-association	1.13	2	1.03
Klebsiella spp.	Sex	1.01	1	1.01
Klebsiella spp.	Age group (years)	1.14	6	1.01
Klebsiella spp.	Region	1.12	41	1.00
Klebsiella spp.	Financial year	1.01	1	1.01
Klebsiella spp.	Month	1.04	11	1.00
P. aeruginosa	Multi-drug resistance	1.05	1	1.02
P. aeruginosa	Healthcare-association	1.16	2	1.04
P. aeruginosa	Sex	1.02	1	1.01
P. aeruginosa	Age group (years)	1.19	6	1.01
P. aeruginosa	Region	1.20	41	1.00
P. aeruginosa	Financial year	1.02	1	1.01
P. aeruginosa	Month	1.09	11	1.00

Table 4: Frequency and incidence rates of *Escherichia coli*, *Klebsiella* species and *Pseudomonas aeruginosa* bloodstream infections by treatment specialty and predisposing risk factors, England, 1 April 2017–31 March 2022

**Characteristics**	***E. coli***		***Klebsiella* spp.**		***P. aeruginosa***	
	**N (%)**	**IR [a] (95% CI)**	**N (%)**	**IR (95% CI)**	**N (%)**	**IR (95% CI)**
**Treatment specialty [b]**	**180,354 (89.3%)**	-	**47,977 (89.3%)**	-	**18,720 (87.6%)**	-
General Medicine	8,615 (26.2%)	23.8 (23.3-24.4)	3,434 (23.1%)	9.5 (9.2-9.8)	1,572 (22.7%)	4.4 (4.1-4.6)
General Surgery	3,862 (11.7%)	24.7 (23.9-25.5)	1,642 (11.1%)	10.5 (10.0-11.0)	515 (7.4%)	3.3 (3.0-3.6)
Geriatric medicine	3,813 (11.6%)	14.6 (14.2-15.1)	986 (6.6%)	3.8 (3.5-4.0)	487 (7.0%)	1.9 (1.7-2.0)
Haematology	1,858 (5.6%)	61.5 (58.7-64.3)	1,004 (6.8%)	33.2 (31.2-35.3)	851 (12.3%)	28.1 (26.3-30.1)
Gastroenterology	1,765 (5.4%)	27.7 (26.4-29.0)	909 (6.1%)	14.3 (13.3-15.2)	232 (3.3%)	3.6 (3.2-4.1)
Oncology	1,352 (4.1%)	52.7 (49.9-55.6)	563 (3.8%)	21.9 (20.2-23.8)	429 (6.2%)	16.7 (15.2-18.4)
Trauma & Orthopaedics	1,277 (3.9%)	9.3 (8.8-9.8)	359 (2.4%)	2.6 (2.3-2.9)	179 (2.6%)	1.3 (1.1-1.5)
Critical Care medicine	1,047 (3.2%)	336.7 (316.6-357.7)	1,191 (8.0%)	383.0 (361.5-405.4)	469 (6.8%)	150.8 (137.5-165.1)
Cardiology	962 (2.9%)	13.2 (12.4-14.1)	488 (3.3%)	6.7 (6.1-7.3)	247 (3.6%)	3.4 (3.0-3.8)
Paediatrics	930 (2.8%)	13.4 (12.6-14.3)	728 (4.9%)	10.5 (9.7-11.3)	305 (4.4%)	4.4 (3.9-4.9)
Urology	849 (2.6%)	27.5 (25.7-29.5)	366 (2.5%)	11.9 (10.7-13.2)	177 (2.6%)	5.7 (4.9-6.7)
Neurology	705 (2.1%)	16.1 (15.0-17.4)	424 (2.9%)	9.7 (8.8-10.7)	188 (2.7%)	4.3 (3.7-5.0)
Nephrology	695 (2.1%)	25.1 (23.3-27.1)	345 (2.3%)	12.5 (11.2-13.9)	170 (2.5%)	6.1 (5.3-7.1)
Accident & Emergency	356 (1.1%)	24.8 (22.3-27.5)	142 (1.0%)	9.9 (8.3-11.7)	51 (< 1%)	3.6 (2.6-4.7)
Respiratory/Thoracic Medicine	178 (< 1%)	1.6 (1.4-1.8)	182 (1.2%)	1.6 (1.4-1.9)	92 (1.3%)	0.8 (0.7-1.0)
Others	4,632 (14.1%)	19.6 (19.0-20.1)	2,084 (14.0%)	8.8 (8.4-9.2)	970 (14.0%)	4.1 (3.8-4.4)
**Predisposing risk factors [c]**						
Urinary catheter inserted or Manipulated	16,136/61,043 (26.4%)	-	4,470/10,734 (41.6%)	-	2,127/4,341 (49.0%)	-
Urinary tract infection treatment	9,394/48,565 (19.3%)	-	1,514/8,186 (18.5%)	-	674/3,312 (20.4%)	-
Prior surgery	8,321/91,377 (9.1%)	-	2,576/20,287 (12.7%)	-	1,151/8,040 (14.3%)	-
Vascular device inserted or Manipulated	3,926/14,324 (27.4%)	-	2,224/4,526 (49.1%)	-	1,266/2,396 (52.8%)	-
Neutrophil count <500	3,701/97,537 (3.8%)	-	1,192/22,160 (5.4%)	-	1,079/8,601 (12.5%)	-
Open wounds or ulcers	2,269/14,563 (15.6%)	-	631/3,485 (18.1%)	-	715/2,328 (30.7%)	-
Hepatobiliary procedure	1,903/29,934 (6.4%)	-	642/7,447 (8.6%)	-	114/2,038 (5.6%)	-
Intubation or extubation	1,225/20,135 (6.1%)	-	1,185/5,839 (20.3%)	-	520/3,053 (17.0%)	-
Prostate biopsy	660/63,954 (1.0%)	-	52/11,023 (< 1%)	-	29/4,401 (< 1%)	-
Diabetic foot ulcer	480/15,024 (3.2%)	-	149/3,631 (4.1%)	-	129/2,351 (5.5%)	-

CI: confidence interval; IR: incidence rate

[a] Incidence rate per 100,000 overnight bed-days.

[b] Hospital-onset cases only.

[c] Only infections between April 2020 and March 2022 are included.

Table 5. Geographical distribution of healthcare-associated and community-associated *Escherichia coli*, *Klebsiella* species and *Pseudomonas aeruginosa* bloodstream infections, England, 1 April 2020–31 March 2022

Region	*Escherichia coli*						*Klebsiella species*						*Pseudomonas aeruginosa*					
	HA			CA			HA			CA			HA			CA		
	n	IR[a]	95% CI	n	IR	95% CI	n	IR	95% CI	n	IR	95% CI	n	IR	95% CI	n	IR	95% CI
North-West	6,445	45.5	44.4-46.6	3,235	32.7	31.6-33.9	1,438	10.1	9.6-10.7	1,362	13.8	13.1-14.5	361	2.5	2.3-2.8	447	4.5	4.1-5.0
North-East and Yorkshire	8,652	50.1	49.0-51.1	4,673	42.1	40.9-43.4	2,070	12.0	11.5-12.5	1,762	15.9	15.2-16.6	539	3.1	2.9-3.4	717	6.5	6.0-7.0
Midlands	9,304	43.6	42.8-44.5	4,736	38.5	37.4-39.6	2,155	10.1	9.7-10.5	1,867	15.2	14.5-15.9	687	3.2	3.0-3.5	920	7.5	7.0-8.0
East of England	5,732	43.7	42.5-44.8	2,350	32.6	31.3-34.0	1,311	10.0	9.5-10.5	1,090	15.1	14.2-16.0	462	3.5	3.2-3.9	528	7.3	6.7-8.0
South-West	5,303	46.8	45.5-48.1	2,362	34.2	32.9-35.6	1,218	10.7	10.2-11.4	857	12.4	11.6-13.3	383	3.4	3.0-3.7	358	5.2	4.7-5.8
London	6,292	34.9	34.1-35.8	3,067	29.5	28.5-30.6	1,724	9.6	9.1-10.0	2,029	19.5	18.7-20.4	544	3.0	2.8-3.3	1,045	10.1	9.5-10.7
South-East	8,287	46.4	45.4-47.4	3,638	41.0	39.7-42.4	1,824	10.2	9.7-10.7	1,599	18.0	17.2-18.9	717	4.0	3.7-4.3	840	9.5	8.8-10.1

CA: Community-associated, CI: confidence interval; HA: Healthcare-associated, IR: incidence rate

[a] Incidence rate of healthcare-associated BSI is per 100,000 overnight bed-days and day admission. Incidence rate of community-associated BSI is per 100,000 population.

Figure 1: Count of episodes where insertion or manipulation of a urinary catheter was a predisposing risk factor for *Escherichia coli*, *Klebsiella* species and *Pseudomonas aeruginosa* bloodstream infections in England by age group, sex and healthcare-association: 1st April 2020 to 31st March 2022

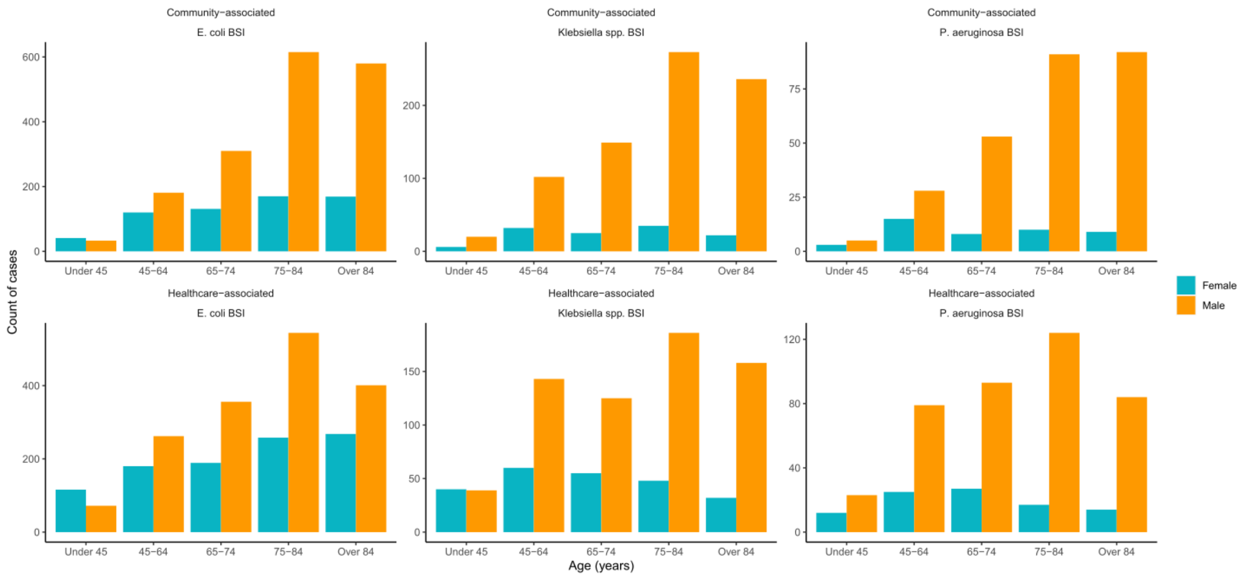

Figure 2: Count of cases where urinary tract infection was the primary focus of infection for *Escherichia coli*, *Klebsiella* species and *Pseudomonas aeruginosa* bloodstream infections in England by age group, sex and healthcare-association: 1st April 2020 to 31st March 2022

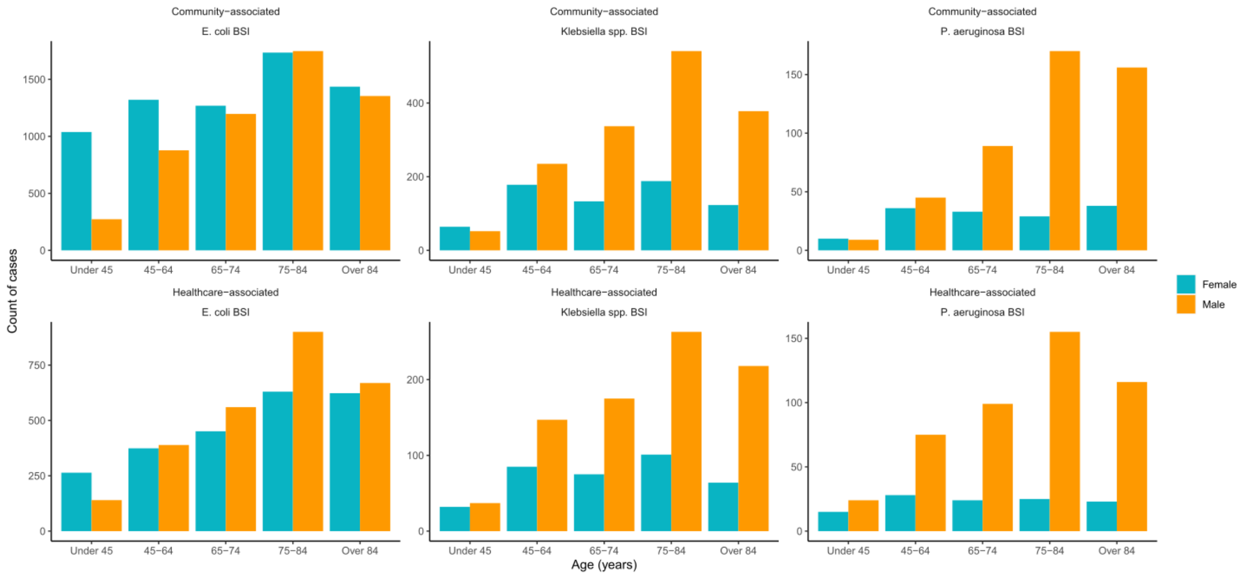

Supplement: Supplementary Material [file 24-00430_NSONWU_Supplement.pdf]
